# Popular justice and territorial resistance in the Peruvian Andes: the case of Huanta

**DOI:** 10.3389/fsoc.2025.1602160

**Published:** 2025-05-22

**Authors:** Fernando Gari Huayhua Lévano, Bruno Kadafi Cardenas Morales, Rubén Ñaupari Molina, Saríah Fanny Oré Gálvez, Alex Quispe Quispe, Ivet Danitza Coronado Illanes, Fernando Félix Huayhua Aguirre, Amilcar Tacuri Gamboa, Jhon Ivan Ramos Malpica

**Affiliations:** ^1^Escuela Profesional de Ingeniería y Gestión Ambiental, Universidad Nacional Autónoma de Huanta, Ayacucho, Peru; ^2^Escuela Profesional de Educación, Especialidad de Ciencias Sociales, Universidad Nacional de San Antonio Abad del Cusco, Cusco, Peru; ^3^Independent Researcher, Huanta, Peru; ^4^Escuela Profesional de Ingeniería de Minas, Universidad Nacional de San Cristóbal de Huamanga, Ayacucho, Peru

**Keywords:** protest radicalization, popular justice, socio-environmental conflict, historical memory, self-governance, plebeian mobilization, Huanta, Peruvian Andes

## Abstract

Huanta, known as “The Emerald of the Andes,” is a historical epicenter of radical mobilization in southern Peru, where resistance has emerged against a state perceived as absent and repressive. This study aims to analyze the radicalization of protest through three key events: the 1969 Rebellion opposing educational reforms under Velasco Alvarado’s regime; the burning of the Provincial Prosecutor’s Office in 2022 as an act of popular justice; and the 2024 indefinite strike in the Razuhuillca watershed against mining expansion. Using a qualitative approach based on in-depth interviews, documentary analysis, and thematic coding, this research identifies three dimensions: historical memory of resistance, crisis of state legitimacy, and communal territorial defense. Drawing upon theories of structural, symbolic, necropolitical, and territorial violence, the study explores how multiple forms of exclusion intersect to produce radicalized collective action. The findings reveal that radicalization in Huanta is rooted in intergenerational memory, community cohesion, and self-governance, rather than spontaneous impulses. This suggests that plebeian protest is not an anomaly but a structural response to exclusion and state violence, offering insights into broader dynamics of resistance and community governance in the Peruvian Andes.

## Introduction

Huanta, known as “The Emerald of the Andes,” is a city located in the Ayacucho region of Peru. Situated at 2,627 m above sea level, its inter-Andean geography and strategic position between the highlands and the jungle make it a key node of territorial, economic, and cultural articulation (Crisóstomo Meza, 2011). However, beyond its geopolitical relevance, Huanta represents a space historically marked by an ambivalent relationship with the Peruvian state: selective presence, violent repression, and a long-standing history of institutional exclusion (Seoane, 2006; Paredes et al., 2006).

This study contributes to expanding new theoretical approaches to understanding violence by analyzing how historical memory, territorial disputes, and legitimacy crises intertwine with structural, symbolic, necropolitical, and territorial forms of violence. In doing so, it seeks to offer broader insights into the ways violence shapes grassroots mobilization and community governance dynamics.

The city has witnessed multiple episodes of radical mobilization, which must not be understood as spontaneous reactions but rather as expressions of a political rationality built from below. One of the foundational milestones in this culture of resistance is the 1969 Rebellion, during which students and peasants confronted the educational reform project of Velasco Alvarado’s military regime. The violent repression unleashed against this protest consolidated a symbolic repertoire of struggle that has been transmitted intergenerationally (Crisóstomo Meza, 2011). This insubordinate memory continues to shape contemporary narratives of protest, functioning as a legitimizing force for new struggles (Bolo-Varela, 2024).

Decades later, in November 2022, Huanta once again became the epicenter of a radical protest: the burning of the Provincial Prosecutor’s Office, in reaction to the judicial handling of a homicide case that the population perceived as emblematic of structural impunity. This act of popular justice reflects not only a rupture with formal judicial institutions but also the emergence of parallel normative systems in contexts of state delegitimization (Fuentes-Nieva and Nelli Feroci, 2017; Osorio-Rozo and Olarte-Cancino, 2023).

Most recently, the indefinite strike in the Razuhuillca watershed in August 2024 stands as a new expression of territorial resistance. In response to mining expansion without prior consultation, communal organizations articulated a discourse centered on the defense of water, common goods, and self-determination. This conflict aligns with the eco-territorial turn described by Svampa (2019), wherein territories are defended not only for their material value but also as vital spaces of life and sovereignty.

Although these events arise from distinct historical moments, this study analyzes them as manifestations of a structural pattern of confrontation with the state. Drawing from Galtung’s (1969) theory of structural violence, Mbembe’s (2003) notion of necropolitics, Bourdieu’s (1991) concept of symbolic violence, and Santos’s (2002a,b) framework of legal pluralism, this research seeks to understand how various forms of violence intersect in the radicalization of protest in Huanta. Additionally, Habermas’s (1975) theory of legitimation crisis offers a valuable lens to examine how institutional breakdowns contribute to the emergence of alternative governance structures and communal justice mechanisms.

By articulating these theoretical perspectives, the study explores how processes of structural exclusion, territorial dispossession, and epistemic marginalization converge to produce conditions of radicalization. Furthermore, it elucidates how communal actors in Huanta construct counter-hegemonic discourses and practices that challenge the authority of the state while asserting their right to self-determination and justice.

The radicalization of protest in Huanta refers to forms of collective action that arise in response to perceived limit situations—such as corruption, impunity, or threats to communal sovereignty—and are characterized by a rupture with traditional institutional channels, mass participation, and the adoption of direct confrontation tactics, including total strikes, road blockades, economic shutdowns, and, in extreme cases, symbolic acts of popular justice such as the destruction of state infrastructure. This form of protest does not stem from individual or spontaneous logic, but rather from a territorial political rationality that articulates historical memory, community cohesion, and structural disobedience.

### Theoretical framework

Understanding the radicalization of protest in Huanta requires an integrated conceptualization of violence that transcends traditional notions of direct physical repression. Rather than isolated phenomena, violence in Huanta manifests as a deeply intertwined network of structural, symbolic, necropolitical, territorial, legal, and epistemic forms, each reinforcing the others and rooted in the historical and everyday experiences of marginalization.

At the foundation lies structural violence, theorized by Galtung (1969), which manifests through systemic inequalities embedded in social, political, and economic institutions. In Huanta, decades of selective state presence, denial of quality education, healthcare deficiencies, and judicial inaction have produced cumulative harms that remain largely invisible yet deeply felt. The imposition of top-down educational reforms in 1969, sparking rebellion among students and peasants, exemplifies how structural violence delegitimized local realities in favor of centralized models of governance.

Building upon these fractures, symbolic violence, conceptualized by Bourdieu (1991), operates by legitimizing domination through the internalization of social hierarchies. In Huanta, rural and Indigenous knowledge systems were systematically marginalized under national policies that privileged urban, Western-centric models. Communities internalized feelings of exclusion and inferiority, reinforcing the silent reproduction of inequalities across generations. This symbolic violence normalized the abandonment experienced under structural frameworks.

As these processes deepen, necropolitics, as defined by Mbembe (2003), emerges in contexts where populations are rendered disposable. In Huanta, extractive incursions into the Razuhuillca watershed without consultation or safeguards expose communities to environmental devastation and existential threats. Necropolitical governance thus intersects with earlier structural and symbolic violences, converting abandonment into an active politics of death over territories and populations deemed sacrificial.

Territorial violence, elaborated by Svampa (2019), directly links these dynamics to the transformation and degradation of physical spaces. In Huanta, the defense of water is not merely an environmental concern but a defense of collective identity and life itself. The destruction of sacred ecological sites under mining interests dismantles both the material and symbolic foundations of communal existence, illustrating how territorial dispossession is not separate from necropolitical exposure but an extension of it.

This web is reinforced by legal violence, understood through Santos’s (2002a,b) concept of legal pluralism. When the formal legal system ceases to guarantee justice—as evidenced by the burning of the Provincial Prosecutor’s Office in 2022—communities construct alternative normative frameworks. Popular justice in Huanta arises not as disorder but as a rational, organized response to structural exclusion and the erosion of institutional credibility.

Finally, epistemic violence, theorized by Walsh (2007) and Rivera Cusicanqui (2010), pervades these processes by devaluing Indigenous knowledge systems and communal epistemologies. In Huanta, the sidelining of ancestral environmental practices and communal decision-making structures reflects not only material domination but also a cognitive erasure that denies alternative ways of being and resisting.

These forms of violence are not isolated; they are mutually reinforcing and co-constitutive. Structural inequalities normalize symbolic subjugation; symbolic exclusion paves the way for necropolitical abandonment; necropolitical exposure unfolds through territorial dispossession; legal violence silences community-driven justice; and epistemic violence erases the very knowledge systems capable of sustaining alternative futures. In Huanta, radical protest emerges as a historically situated, rational strategy of survival and dignity, responding to the layered violences that structure everyday life in the Andes.

Thus, analyzing the radicalization of protest in Huanta requires not a fragmented view of violence, but an integrated theoretical lens capable of capturing the interwoven, cumulative, and historically grounded nature of exclusion and resistance.

## Methodology

### Approach and design

This study adopts a qualitative approach aimed at understanding the processes of protest radicalization in the district of Huanta, Ayacucho. It focuses on three pivotal events: the 1969 Rebellion, the burning of the Public Prosecutor’s Office in 2022, and the indefinite strike in the Razuhuillca watershed in 2024. These events were selected for their significance in the historical memory and ongoing conflict dynamics of the region.

The research is framed within an exploratory qualitative design (Denzin and Lincoln, 2011), suitable for examining complex and evolving phenomena in under-researched contexts such as the Andean region. This approach acknowledges the situated nature of knowledge and the agency of subaltern actors, recognizing their capacity to construct alternative meanings and practices of justice.

### Data collection and sources

The methodological approach combines documentary analysis, semi-structured interviews, and thematic analysis, supported by triangulation to enhance interpretive validity (Patton, 2002). The data collection process was structured to allow for flexibility and adaptation, ensuring that relevant themes were fully explored.

#### Documentary analysis

Documentary analysis involved a wide range of sources including historical documents, journalistic records, governmental reports, and academic literature. This process aimed to identify dominant and counter-hegemonic narratives related to the selected protest events. Additionally, it allowed the construction of a timeline of critical episodes and the identification of recurring themes related to collective memory, popular justice, and socio-environmental resistance (see Table 1).

**Table 1:** Summary of data sources and analytical techniques.

Source	Description	Analytical technique
Historical documents	Archives on protests and educational legislation	Content analysis
Printed press	National and regional newspapers (El Comercio, La República, RPP)	Discourse analysis
Official reports	Ombudsman’s Office, NGOs, ministerial reports	Documentary triangulation
Interviews	Community leaders, journalists, former officials	Thematic coding

#### Semi-structured interviews

During the preliminary analysis, specific analytical gaps were identified, particularly related to the socio-environmental dimensions of the Razuhuillca conflict and the persistence of popular justice practices. To address these gaps, three additional interviews were conducted in March and April 2024, bringing the total to 15. This decision was made based on the criterion of theoretical saturation (Glaser and Strauss, 1967) (see Table 2).

**Table 2:** Interviewee profiles (*n* = 15).

Code	Reference group	Estimated age	Link to analyzed events	Key role or experience
E01	Huanta community member	60–75 years	Direct participation in the 1969 protest	Local memory and communal leadership
E02	Regional journalist	45–60 years	Media coverage of conflicts (2000–2024)	Media analysis and public discourse
E03	Former local official	65–75 years	Public administration during periods of conflict	State-community relations
E04	Relative of historical leader	30–50 years	Transmission of generational memory	Symbolic continuity and community politics
E05	Territorial activist	35–55 years	Environmental defense and popular justice	Local resistance articulation
E06	Young community member	20–35 years	Participation in recent protests	New generation of mobilization
E07	Alternative communicator	25–40 years	Digital dissemination of conflicts	Counter-hegemonic narrative production
E08	Rural teacher	50–65 years	Education in conflict-affected communities	Education and social memory
E09	Conflict specialist	40–55 years	Academic analysis and fieldwork	Theoretical framework on Andean mobilizations
E10	Traditional community authority	60–75 years	Active role in communal assemblies	Local self-governance practices
E11	Environmental defender (female)	35–50 years	Defense of water in Razuhuillca	Ecological and territorial leadership
E12	University student	20–30 years	Youth activism in 2022 and 2024	Generational rupture and protest innovation
E13	Former urban ronda leader	50–65 years	Urban communal justice post-2000	Popular justice practices
E14	NGO technician	30–45 years	Involvement in extractive conflicts	Documentation of human rights violations
E15	Local Quechua-language reporter	35–50 years	Testimony in community media	Cultural and multilingual advocacy

#### Thematic coverage of the cases

The fifteen interviews conducted addressed all three key events analyzed in this study: the 1969 Rebellion, the burning of the Provincial Prosecutor’s Office in 2022, and the indefinite strike in the Razuhuillca watershed in 2024. These events represent critical milestones in local collective memory and are referenced by diverse social actors, regardless of age, profession, or political background.

The thematic transversality achieved in the interviews allowed for the establishment of intergenerational links, enhancing the comparative analysis across contexts and strengthening the theoretical saturation reached.

While the emphasis on specific events varied according to generational profiles and social roles, all participants referred explicitly to the three core episodes, suggesting that these events function as symbolic repertoires that frame ongoing protest dynamics.

Additionally, other minor events were mentioned during the interviews but were not included in this analysis to maintain a precise narrative corpus focused on the three central episodes. This decision follows a logic of selective coding aimed at enhancing coherence and interpretative rigor.

### Data analysis

The qualitative analysis followed the thematic analysis approach proposed by Braun and Clarke (2006), enabling the identification of meaningful patterns within the corpus of interviews and documents. This analysis was conducted in three articulated phases:

1. Open Coding: Initial identification of 47 codes from interviews and documentary sources. These codes included categories such as memory of protest, structural impunity, state abandonment, defense of water, criminalization of protest, and communal honor.
2. Axial Coding: Reorganization of the 47 initial codes into 12 final thematic categories, structured around three analytical dimensions:

- Historical memory and persistence of mobilization.
- Crisis of state legitimacy and popular justice.
- Socio-environmental conflict and territorial defense.

3. Selective Coding: Integration of categories into a coherent analytical framework using Atlas.ti 23 software. This step facilitated the visualization of interrelated concepts through the generation of semantic networks.

These 12 thematic categories—collective memory, communal autonomy, popular justice, youth resistance, eroded state legitimacy, parallel systems of justice, protest criminalization, territorial dispossession, symbols of resistance, inherited repertoires, anti-extractivist discourse, and water defense—were identified as central to understanding the processes of radicalization.

The heterogeneity of responses was particularly evident when comparing interviewee profiles. Elderly community members emphasized the 1969 Rebellion and the transmission of political memory, while young leaders focused on contemporary environmental conflicts and new organizational forms. Journalists and former officials highlighted institutional breakdown and the erosion of state legitimacy.

To ensure coherence and validity, the integration of multiple techniques—open and axial coding, semantic visualization, and source triangulation (interviews, press, and official documents)—was essential. The use of Atlas.ti 23 software allowed for the creation of a semantic network of codes, revealing the most relevant thematic interconnections.

### Network of emerging codes

During the coding process, a semantic network was constructed to illustrate the relationships between the main categories identified. This network revealed two central nodes—“crisis of state legitimacy” and “territorial resistance”—which intersect and articulate the three key events analyzed in this study. The connections among categories such as collective memory, popular justice, structural impunity, and water defense demonstrate the interconnectedness of historical, political, and environmental dimensions of protest in Huanta.

**Figure 1:**
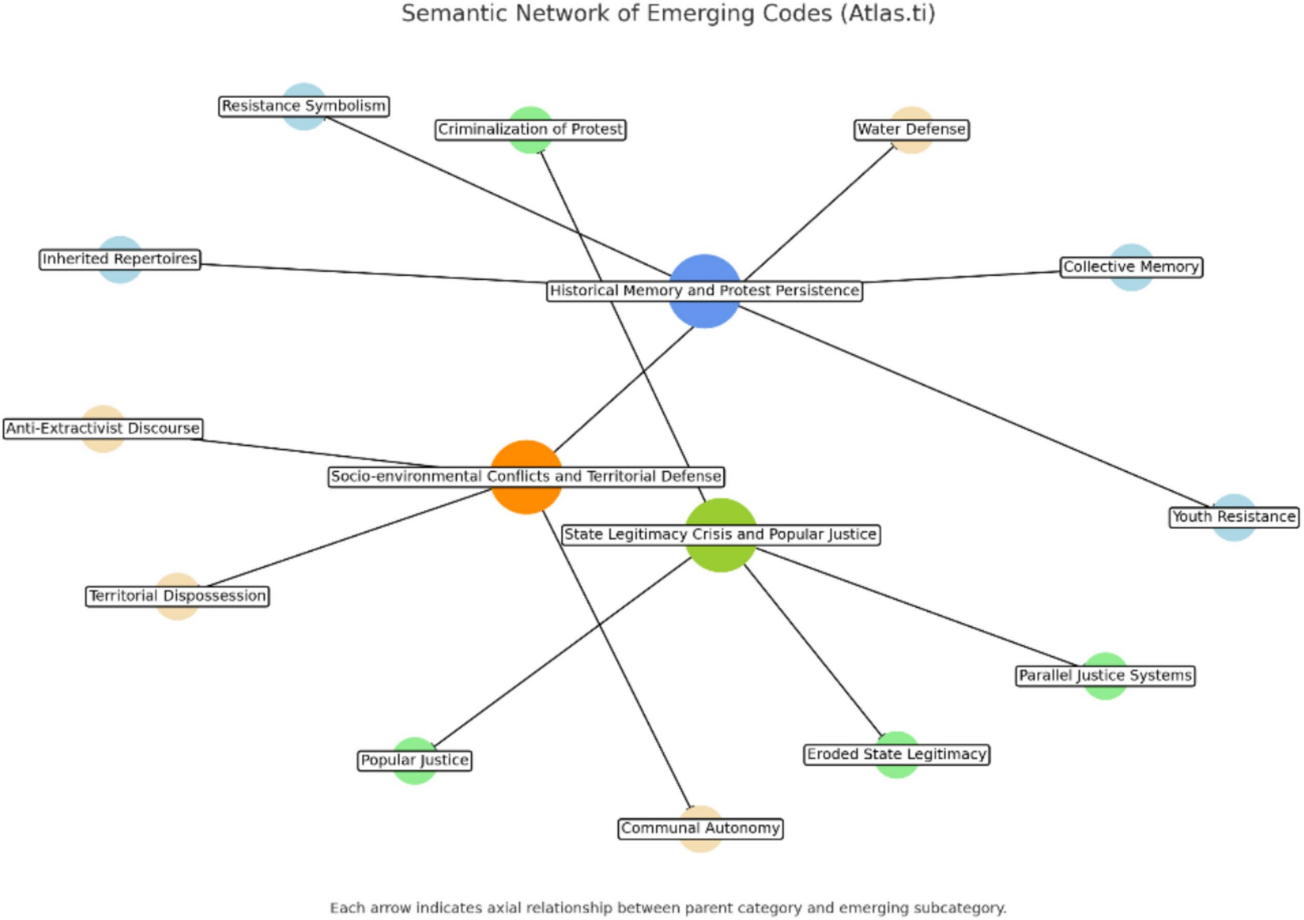
Semantic network of emerging codes generated with Atlas.ti 23.

As visualized in Figure 1, the network structure supports the interpretation of radicalization as a process rooted in overlapping experiences of exclusion, resistance, and territorial self-determination.

### Cross-validation, narrative consistency, and ethical considerations

The methodological triangulation process revealed a notable consistency across various sources analyzed: oral testimonies, journalistic records, and community documents. These sources provided convergent accounts of the events under study, enhancing the interpretive validity of the findings.

Institutional documents and official statements, although reviewed, often emerged belatedly or reactively. Instead of directly contradicting the community narratives, they often highlighted the absence of the State during critical moments of protest. This convergence reinforces the coherence of the study’s analytical framework.

Despite this coherence, a logic of permanent critical contrast was maintained, identifying omissions, silences, and nuances among narratives. This approach ensured that the analysis was not overly simplistic or romanticized, but rather engaged in a reflexive and critical process of interpretation.

### Ethical considerations

The research adhered to ethical principles of confidentiality, informed consent, and cultural sensitivity, following approval by the Research Ethics Committee of the Universidad Nacional Autónoma de Huanta. To protect participants’ identities, alphanumeric codes (e.g., E01, E02) were used and profiles were drafted to prevent public identification. Informed consent was obtained prior to all interviews, ensuring participants’ voluntary involvement and their right to withdraw at any time. Special attention was paid to testimonies provided by descendants of historical actors, acknowledging the epistemic value of inherited memory while safeguarding familial privacy. Narratives were handled as socially situated constructions rather than absolute truths, with efforts made to avoid harmful recontextualization. Preliminary findings were presented to participants for validation, promoting dialogic feedback and epistemic horizontalism. Ethical considerations were integrated throughout the research process, reflecting a commitment to non-revictimization, cultural sensitivity, and participatory return of findings.

## Results

The findings of this research shed light on how the radicalization of protest in Huanta is shaped by a combination of historical memory, a crisis of state legitimacy, and socio-environmental conflicts. Thematic analysis conducted with the support of Atlas.ti 23 software revealed a complex network of interrelated categories. As shown in Figure 1, the most relevant thematic interconnections are structured around two central nodes: “crisis of state legitimacy” and “territorial resistance.” These nodes are articulated through categories such as collective memory, popular justice, structural impunity, and water defense, which demonstrate how protest radicalization emerges from intersecting experiences of exclusion, resistance, and self-determination.

The visualization of these relationships (Figure 1) provides a comprehensive analytical map that supports the interpretation of radicalization as a structured and multi-dimensional phenomenon.

### Historical memory and the persistence of mobilization

The persistence of mobilization in Huanta is deeply rooted in the intergenerational transmission of historical memory. This dynamic can be interpreted through Tarrow’s (1998) notion of protest repertoires, where collective memories act as templates for action that are reactivated in response to new forms of structural injustice (Galtung, 1969).

A descendant of a historical leader recounted:

“My father always told me how they marched for free education in 1969. It wasn’t just about learning; it was about dignity. Now it’s our turn to continue that struggle” (E04, Relative of historical leader).

This testimony illustrates the updating of a dignity repertoire, where memory becomes a political resource to confront new manifestations of structural violence, such as educational exclusion and state neglect. Similarly, a community member emphasized:

“My grandparents faced the Army, not out of violence but for justice. We learned that from a young age” (E01, Huanta community member).

These narratives reveal how the intergenerational transmission of insurgent memory serves as a form of resistance against the state’s symbolic imposition of official narratives, aligning with Bourdieu’s (1991) concept of symbolic violence. In this context, reviving and maintaining collective memory resists not only symbolic domination but also challenges the deeper processes of epistemic violence, as theorized by Walsh (2007), whereby communal knowledge systems and historical experiences are marginalized or erased.

The ritual repetition of marches, the use of historical symbols, and the reactivation of ancestral slogans—as several participants described—reinforce the continuity of a symbolic and epistemic conflict against a state that has historically sought to devalue and delegitimize communal epistemologies.

### Crisis of legitimacy and popular justice

The legitimacy crisis in Huanta is expressed through widespread perceptions of state neglect and corruption. This disenchantment has led to the adoption of popular justice mechanisms, which communities view as rational responses in contexts where formal institutions are considered ineffective or biased (Serulnikov, 2019; Santos, 2002a,b). The burning of the Provincial Prosecutor’s Office in 2022 exemplifies this phenomenon, signaling not only popular disenchantment but also the emergence of alternative justice structures grounded in communal legitimacy.

Interviewees described this act not as one of hatred, but as a response to persistent judicial inaction. One participant explained, “They never do anything. Murderers and corrupt people are free, while we keep suffering.” Others emphasized that “if we do not protect ourselves, no one will,” pointing to the rise of urban patrols and community-based justice initiatives in the absence of effective state protection.

Popular justice is thus understood as a legitimate form of self-defense rather than lawlessness. This radical shift can be interpreted through the lens of structural violence (Galtung, 1969), where the systematic denial of access to justice perpetuates harm and reinforces social exclusion. Simultaneously, the creation of communal justice systems reflects the principles of legal pluralism proposed by Santos (2002a,b), recognizing the coexistence of alternative normative frameworks where the state’s authority collapses.

As expressed by one community member, “We wanted them to see that we no longer trust them, that if they do not provide justice, we will.” These testimonies demonstrate that justice is reinterpreted not as the exclusive domain of the state, but as a collective right activated through communal agency when institutional mechanisms fail.

**Figure 2:**
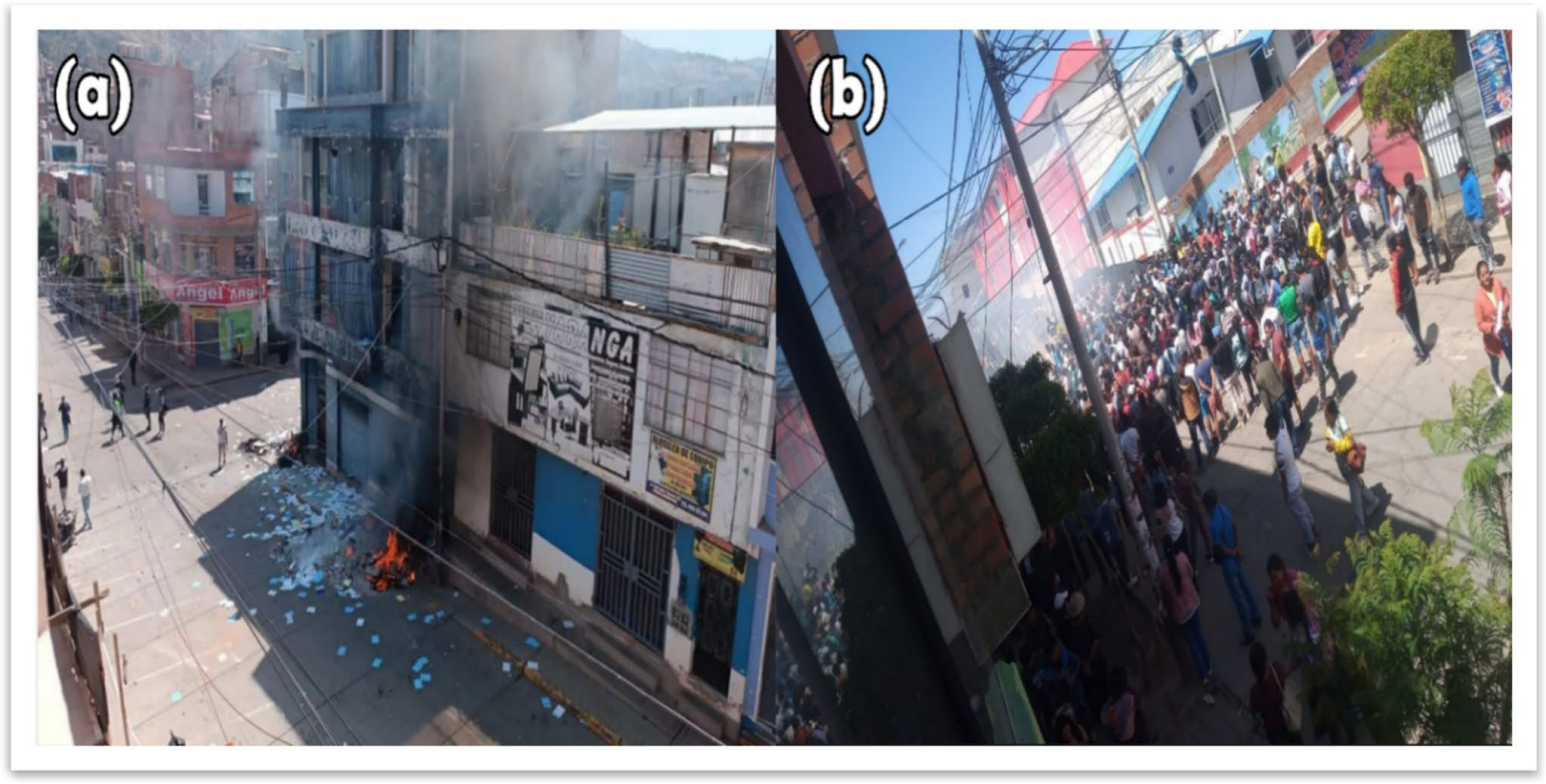
Popular justice and state legitimacy crisis in Huanta. **(a)** Burning of the Provincial Prosecutor’s Office in 2022 as a symbolic act of protest. **(b)** Gathering of community members and youth following the event.

This shift also aligns with Bourdieu’s (1991) notion of symbolic violence, highlighting how institutional failure not only produces a material legitimacy vacuum but also delegitimizes alternative community forms of governance, which are actively reasserted through acts of protest and popular justice (see Figure 2).

### Socio-environmental conflict and territorial defense

The socio-environmental conflict in Razuhuillca (2024) marks a new chapter in Huanta’s broader struggle for territorial sovereignty. Aligned with Svampa’s (2019) concept of the eco-territorial turn, this mobilization reveals how environmental defense is deeply intertwined with political, cultural, and existential demands.

Participants frequently described water as not just a resource but a symbol of life and identity. One environmental defender stressed that “water is life” and that defending it means protecting the community’s future. These sentiments were echoed by others who saw the mining threat as an existential one, emphasizing that “we would rather die defending water than live without it.” Such statements reveal that the stakes of the conflict go beyond ecological concerns—they represent a defense of communal ways of life.

The experience with mining in other regions has shaped a collective refusal to accept extractive activities, which are perceived not only as economic threats but also as acts of territorial violence (Svampa, 2019)—a systematic attack on communal identities, rights, and spaces of life. The communities’ articulation of protest slogans such as “Water is not a commodity” reflects this broader understanding that defending the territory is equivalent to defending existence itself.

Furthermore, the state’s complicity in promoting extractive expansion without prior consultation exacerbates the exposure of Andean populations to environmental degradation and disposability, aligning with Mbembe’s (2003) notion of necropolitics. The denial of access to clean water and the deterioration of ecosystems are not merely accidental consequences but active forms of governance that selectively sacrifice marginalized communities in the name of economic interests.

The population’s experience with broken negotiation processes has further fueled disillusionment. As one young university student put it, “They promised dialogue, but they never kept their word.” Others linked the current protest to historical struggles, stating that “before, we protested for education. Now it’s for water. But the root is the same: we always have to fight to be heard.”

**Figure 3:**
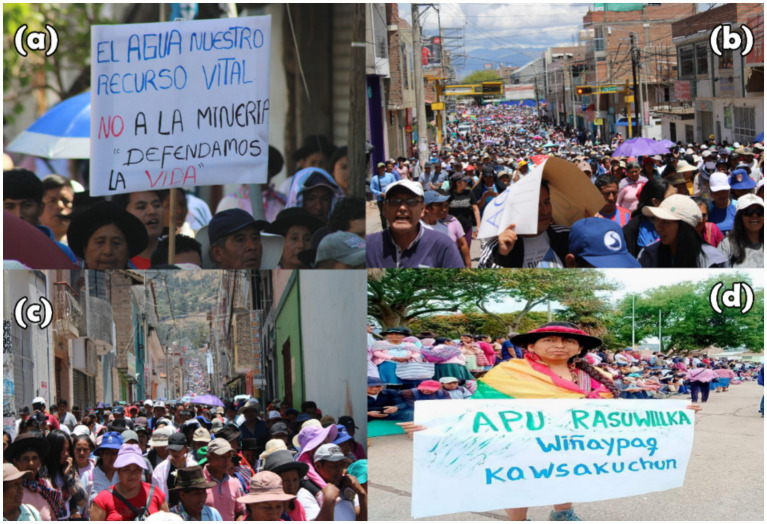
Socio-environmental conflict and territorial defense in Huanta. **(a)** Protest banner against mining: “Water is our vital resource, no to mining.” **(b)** Massive march for water rights. **(c)** Mobilization of Andean communities through central Huanta. **(d)** Woman holding sign in Quechua: “Apu Razuwillka, Wiñaypaq Kawsakuchun” (“Lord Razuhillca, may you live forever”).

This articulation of environmental and cultural resistance underscores the convergence of ecological defense with broader political claims for dignity, sovereignty, and communal survival (see Figure 3).

This figure illustrates the scale of mobilization in Razuhuillca, with entire communities participating in massive marches and articulating a discourse based on the defense of life and water. The artistic and symbolic expressions reflect the profound cultural bond with the territory and nature.

### Cross-validation of findings

The validity of this study’s findings was strengthened through qualitative data triangulation, a fundamental strategy in exploratory research with an interpretive approach (Patton, 2002). This procedure involved the systematic comparison of narratives derived from semi-structured interviews with documentary records and journalistic sources related to the three selected events. Such triangulation enabled a robust analysis that acknowledges the complexity of the processes of radicalization in Huanta.

In the case of the 1969 Rebellion, testimonies from descendants of student leaders and community organizers were cross-referenced with prior academic publications and contemporary press reports. This triangulation confirmed not only the occurrence of the events but also the ways in which they have been re-signified across generations. The persistence of protest repertoires based on dignity and social justice reveals the presence of a deep historical consciousness among the population (Tarrow, 1998; Almeida and Johnston, 2006a,b). One relative of a historical leader explained that “we cannot forget what happened in ‘69. It is like an open wound. That pain and pride are what keep us fighting,” reinforcing the symbolic importance of intergenerational memory.

Regarding the burning of the Provincial Prosecutor’s Office in 2022, the analysis drew on official statements, local news reports, and interviews with journalists who covered the incident. The convergence of these sources validated the hypothesis that this was an act of popular justice broadly legitimized by various sectors of the community. According to a local Quechua-language reporter, “burning the Prosecutor’s Office was not just an act of anger; it was a declaration that we are tired of being ignored.” These accounts underscore the widespread perception of institutional failure and the emergence of alternative legitimacy mechanisms when the justice system is perceived as inaccessible or corrupt (Santos, 2002a,b).

In the Razuhuillca strike of 2024, interviews with community leaders and environmental defenders were compared with NGO reports, communal assembly records, and regional press coverage. This revealed a consistent narrative centered on the absence of meaningful dialogue mechanisms and the centrality of water defense in framing the conflict (Mora Castillo and Álvarez Manríquez, 2021; Espinosa, 2021). As one activist emphasized, “we fight for water because without it, we have nothing. It’s our life, our culture, our future,” revealing how environmental struggles are inseparable from cultural and existential dimensions. The eco-territorial perspective described by Svampa (2019) finds clear resonance in these testimonies, which portray resistance as a defense of both life and communal sovereignty.

Furthermore, the triangulation process was crucial for identifying contradictions and tensions within the community. While most interviewees endorsed the legitimacy of the protest actions, others expressed concern about potential escalation and the limitations of popular justice. For instance, a traditional community authority stated that “we have tried to negotiate so many times, but they never listen. This is why we must defend our territory ourselves,” illustrating both the frustration with formal processes and the perceived necessity of direct action.

The cross-validation approach applied in this research reduced interpretive bias, helped refine emerging categories, and enhanced the robustness of the findings by anchoring them in a plurality of convergent sources. The integration of oral testimonies, journalistic reports, NGO documents, and governmental records ensured that the analysis remained comprehensive, balanced, and grounded in diverse perspectives.

## Discussion

The findings of this study confirm that the radicalization of protest in Huanta results from the convergence of historical, institutional, and socio-environmental factors that have shaped a consistent pattern of mobilization. This section discusses these results through the lens of critical theory, connecting them to broader processes of social resistance and state-society relations in Latin America.

### Historical memory, legitimacy crisis, and popular justice

The process of protest radicalization in Huanta is rooted in the interplay of three fundamental dimensions: historical memory, institutional legitimacy crises, and the emergence of popular justice mechanisms. This phenomenon is evident through three key events: the 1969 Rebellion, the burning of the Provincial Prosecutor’s Office in 2022, and the indefinite strike in the Razuhuillca watershed in 2024. Each of these episodes should not be understood as isolated incidents but as manifestations of a structural pattern of confrontation consolidated over decades.

From a theoretical perspective, the intergenerational transmission of resistance memory acts as a symbolic device that legitimizes new struggles by appealing to historical genealogies of vindication. According to Tarrow (1998), protest repertoires function as symbolic frameworks that adapt and transform in response to changing contexts of injustice. In Huanta, the 1969 Rebellion constitutes a reference point that not only inspires but also justifies the adoption of increasingly radical protest strategies. This phenomenon aligns with the notion of strategic continuity proposed by Almeida and Johnston (2006a,b), who argue that the memory of past struggles is updated and re-signified to sustain new episodes of mobilization.

The legitimacy crisis in Huanta is expressed through widespread perceptions of state abandonment and institutional corruption. According to Habermas (1973), institutions lose normative authority when they fail to meet societal expectations for justice and protection. In this regard, the persistent neglect of Andean communities—particularly in access to justice and public services—exemplifies what Galtung (1969) describes as structural violence, whereby institutional frameworks perpetuate harm not through direct force but through systematic omission and inequality. This legitimacy deficit has been exacerbated in Huanta due to perceptions of impunity and corruption within the judicial system, manifested in events such as the burning of the Provincial Prosecutor’s Office in 2022. This act of popular justice reflects not only accumulated frustration with a system perceived as ineffective but also the affirmation of alternative legitimacy mechanisms based on community principles of justice.

The adoption of popular justice mechanisms in Huanta should not be interpreted as a spontaneous or irrational response but rather as an organized practice that responds to institutional failure to guarantee fundamental rights. Santos (2002a,b) argues that, in contexts of structural exclusion, communities develop alternative normative systems that challenge the state’s exclusivity in the administration of justice. In Huanta, popular justice emerges as a legitimate mechanism of self-defense against the absence of effective state guarantees.

Furthermore, Habermas’s (1984) Theory of Communicative Action provides a useful framework for understanding how the lack of effective deliberation and dialogue channels exacerbates radicalization. The inability to establish genuine mechanisms of interlocution with the State generates antagonisms that drive communities to adopt direct confrontation strategies. This phenomenon is evident in the burning of the Provincial Prosecutor’s Office, an act symbolizing both despair at the lack of institutional response and the reaffirmation of community autonomy.

On the other hand, the exclusion of alternative justice mechanisms by formal institutions constitutes a form of symbolic violence. Bourdieu (1991) uses this concept to refer to the imposition of meanings that delegitimize or render invisible other forms of knowledge or political action. In this context, the criminalization of popular justice by the State reinforces a hierarchical order that perpetuates the exclusion of Andean communities. Testimonies gathered during this research demonstrate that such delegitimization is not only rejected but actively contested through communal narratives that affirm autonomy and moral authority.

Likewise, the re-signification of historical memory in Huanta is linked to processes of cultural and political resistance that challenge official narratives imposed by the State. Rivera Cusicanqui (2010) argues that insurgent memory is an essential resource for constructing collective identities in resistance, particularly in contexts of persistent coloniality. In Huanta, the evocation of the 1969 Rebellion continues to be a crucial symbolic reference for legitimizing contemporary struggles.

The interviews conducted during this research confirm this dynamic. One participant stated:

“My father always told me how they marched for free education in 1969. It wasn’t just about learning; it was about dignity. Now it’s our turn to continue that struggle.” (E04, Relative of historical leader).

This testimony illustrates not only the continuity of memory but also the symbolic violence resisted through intergenerational transmission of struggle. Similarly, another interviewee remarked:

“We wanted them to see that we no longer trust them, that if they do not provide justice, we will seek it ourselves.” (E15, Quechua-language reporter).

This voice echoes the exhaustion produced by structural violence—the institutional abandonment that leads to the reactivation of popular justice as a form of survival and legitimacy.

In summary, the radicalization of protest in Huanta must be understood as a complex process where historical memory, legitimacy crises, and alternative justice mechanisms converge with structural and symbolic violence. This pattern of resistance, although deeply rooted in the past, is continuously updated and transformed to respond to new conditions of exclusion and repression.

### Socio-environmental conflict and territorial defense

The socio-environmental conflict in Huanta, particularly the indefinite strike in the Razuhuillca watershed in 2024, illustrates how territorial defense becomes a central axis of mobilization in response to extractivist policies and institutional exclusion. This conflict aligns with what Svampa (2019) describes as the eco-territorial turn, where social movements articulate environmental defense with political, cultural, and territorial demands.

The struggle for the defense of the Razuhuillca watershed must be understood not only as an ecological issue but as a broader political struggle over sovereignty, autonomy, and the right to self-determination. The interviews conducted reveal that the population perceives mining activities as a direct threat to their livelihoods, cultural practices, and the continuity of communal life.

The prioritization of extractivist interests over communal rights is an example of Galtung’s (1969) concept of structural violence, which highlights how institutional frameworks perpetuate inequality and exclusion by systematically privileging corporate interests. In Huanta, mining concessions were granted without prior consultation or meaningful participation from local communities. This exclusionary process is perceived as a violation of basic rights, particularly concerning access to water and land—resources that are essential for agricultural production and cultural reproduction.

From a necropolitical perspective, Mbembe (2003) argues that state power operates through the control over life and death. In Huanta, the degradation of ecosystems and contamination of water sources reflect how extractivist policies render specific populations expendable. This resonates with what Gutiérrez Aguilar (2017) describes as the politics of death, where economic growth is prioritized at the expense of communal survival. In this framework, environmental destruction is not merely an unintended consequence, but a biopolitical strategy that exposes certain communities to death or disposability.

Furthermore, the symbolic violence identified by Bourdieu (1991) is evident in the state’s imposition of development narratives that delegitimize alternative epistemologies rooted in communal sovereignty and ecological care. By framing mining projects as essential for economic progress, official discourse marginalizes communal decision-making processes and disregards local knowledge systems. This discursive violence reinforces a hierarchical understanding of development that systematically excludes rural and Indigenous communities from participation.

A crucial dimension to highlight is territorial violence, as theorized by Svampa (2019). Territorial violence refers to the systematic transformation of spaces through extractive activities that displace, fragment, and erode communal structures of life. In Huanta, territorial violence is not limited to environmental degradation; it extends to the symbolic and cultural annihilation of communal identities rooted in the land. The testimonies of protestors, such as those declaring “Water is life. Without it, we cease to exist” (E11) or “We would rather die defending water than live without it” (E07), reveal how territorial violence threatens not just resources but the ontological existence of the community itself. Thus, defending Razuhuillca is both an ecological and existential act of resistance against the violent restructuring of their territory.

Santos’s (2002a,b) concept of abyssal thinking further illustrates how state frameworks systematically render non-Western normative systems invisible or illegitimate. In Huanta, the rejection of communal governance mechanisms and the dismissal of their concerns regarding water and land rights exemplify this exclusionary logic. According to Santos and Sena Martins (2020), such abyssal thinking denies the legitimacy of alternative knowledge systems, thus perpetuating inequality and epistemic violence.

Moreover, effective conservation strategies often depend on integrating educational frameworks that empower local communities to actively engage in the protection of their natural resources. Experiential environmental education, as applied in previous research on the Huaper Wetland, demonstrates how participatory approaches can enhance ecological awareness and promote sustainable practices (Cardenas Morales et al., 2025). This model, grounded in constructivist learning theories, aligns with broader struggles for territorial autonomy by emphasizing community agency and ecological stewardship.

The Razuhuillca watershed strike demonstrates how communal actors articulate their struggles through discourses that challenge the extractivist model and assert alternative frameworks of governance. As noted by Khare (2018), resistance globalization involves connecting local struggles to broader discourses of human rights, environmental justice, and decoloniality. The Razuhuillca protest is not only a reaction to immediate threats but also part of a broader genealogy of resistance against extractivist models imposed by the state.

The testimonies gathered from interviews reinforce these theoretical perspectives. A community leader (E11) stated: “Water is life. The miners do not understand that; they only seek profit. We are defending our future.” This sentiment underscores the centrality of water as both a material resource and a symbolic element essential to communal identity and survival.

Similarly, another interviewee (E07) emphasized: “We have already seen how mining destroys lands elsewhere. Here, we will not allow it. I would rather die defending water than live without it.” This statement is not merely rhetorical—it illustrates what Mbembe (2003) theorizes as necropolitics, a form of power in which the state and corporate actors determine which lives are expendable in the name of economic progress. The imposition of extractive projects without consultation, and in territories where water is sacred and survival is fragile, reflects a governance regime rooted in abandonment and selective exposure to death. In this context, radical protest becomes a form of survival against state-sanctioned disposability.

The conflict also highlights the breakdown of dialogue mechanisms. Despite attempts to establish negotiation tables with regional authorities, these efforts were largely ignored, leading to frustration and the escalation of protest tactics. As noted by a university student (E12): “They promised dialogue, but they never kept their word. The negotiation tables are a joke.” This perception aligns with Habermas’s (1984) Theory of Communicative Action, which posits that the failure of dialogue generates antagonisms and pushes marginalized groups towards direct action.

Furthermore, the Razuhuillca conflict has catalyzed the articulation of broader discourses of environmental justice. As Martínez Alier (2014) argues, the environmentalism of the poor emerges from grassroots struggles for survival against dispossession and ecological degradation. This concept resonates with the testimonies of local actors who frame their struggle as both a fight for water and a defense of cultural autonomy.

The articulation of socio-environmental demands with broader frameworks of political and cultural sovereignty reflects the theoretical insights of Svampa (2019), who describes the eco-territorial turn as a paradigm shift in Latin American social movements. This shift involves the construction of counter-hegemonic discourses that challenge both state repression and neoliberal models of governance.

### Connecting threads: the interplay of memory, legitimacy, and territory

The findings of this study demonstrate that the radicalization of protest in Huanta is not a spontaneous phenomenon, but the result of an accumulated and overlapping network of violences—structural, symbolic, necropolitical, territorial, and epistemic—that has shaped a persistent pattern of grassroots resistance. Historical memory, legitimacy crises, and territorial defense do not operate in isolation; rather, they are deeply interwoven dimensions of a broader political process of contestation.

At the core of this process lies a profound legitimacy crisis, where the failure of institutional justice, as theorized by Habermas (1973), fosters the perception that state mechanisms are unresponsive and corrupt. This is exemplified by the 2022 burning of the Provincial Prosecutor’s Office—a response not only to judicial inaction but to structural violence in Galtung’s (1969) terms, where impunity and abandonment become normalized features of governance.

This collapse of institutional trust also reveals a layer of symbolic violence (Bourdieu, 1991), as the state narrative delegitimizes community-led justice systems and frames direct actions as irrational or criminal. Yet, interviews with Huanta’s leaders show that popular justice is framed not as lawlessness but as dignity in action—a reclamation of autonomy in the face of legal marginalization, echoing Santos’s (2002a,b) idea of legal pluralism.

Meanwhile, the defense of the Razuhuillca watershed crystalizes both territorial violence (Svampa, 2019) and necropolitical exclusion (Mbembe, 2003). The advance of extractive projects without consultation transforms communal lands into zones of dispossession, where environmental degradation and the denial of water access become forms of biopolitical control. In this setting, water is not just a natural resource—it becomes a symbolic and existential axis of communal life, as interviewee E07 affirmed: “I would rather die defending water than live without it.”

Historical memory acts as a binding thread that connects past and present grievances. The evocation of the 1969 Rebellion, as transmitted through families and communal storytelling, aligns with Bolo-Varela’s (2024) notion of insubordinate memory—a counter-hegemonic archive that reframes radical protest as continuity, not rupture. As Tarrow (1998) emphasizes, protest repertoires adapt over time, and in Huanta, that adaptation builds upon layers of trauma, repression, and dignity.

However, this convergence also reveals internal tensions. While historical grievances generate strong cohesion, they also produce conflicts over strategy—notably between dialogue and confrontation. Testimonies indicate that those who express dissent toward confrontational methods may experience epistemic marginalization. This dynamic illustrates what Walsh (2007) and Rivera Cusicanqui (2010) define as epistemic violence—the silencing of alternative viewpoints even within spaces of resistance.

In sum, the radicalization of protest in Huanta emerges as a historically rooted, multidimensional, and rational response to layered forms of violence. Rather than spontaneous or irrational, it represents a conscious political strategy anchored in memory, territorial sovereignty, and popular justice, all shaped by the structural exclusions of the state and global extractivism.

### Broader implications for the study of social mobilization in Latin America

The findings of this study reveal how the radicalization of protest in Huanta reflects broader patterns of social mobilization across Latin America. While the specific historical, cultural, and territorial dynamics of Huanta are unique, they resonate with struggles occurring throughout the region, especially where state absence, repression, and extractivist policies generate organized grassroots responses.

According to Tilly (2003), social movements often emerge from state incapacity to address popular grievances. In Huanta, the state’s inability to reconcile extractivist interests with communal sovereignty has generated crises of legitimacy, fueling radicalization. This dynamic is not unique to Peru but is prevalent across the Andes, where extractivist policies frequently clash with Indigenous and peasant communities’ claims to land and resources (Svampa, 2019; Escobar, 2008).

The persistence of popular justice mechanisms and communal governance in Huanta aligns with Santos’s (2002a,b) concept of legal pluralism, which highlights the coexistence of alternative normative frameworks in contexts where official legal systems are perceived as ineffective or unjust. This phenomenon echoes broader struggles across Latin America, where Indigenous and Afro-descendant communities construct their own normative orders to contest state and corporate interventions (De la Cadena, 2015; Coulthard, 2014).

Furthermore, the articulation of local struggles with transnational networks reflects an increasingly interconnected pattern of resistance. As Khare (2018) describes, “resistance globalization” enables marginalized actors to frame their struggles within broader discourses of human rights, environmental justice, and decoloniality. This process enhances the legitimacy of local demands and reinforces their claims to sovereignty and territorial autonomy.

The conceptualization of violence as a multifaceted phenomenon—encompassing structural, symbolic, territorial, and epistemic dimensions—provides valuable insights into the complexity of contemporary social mobilizations. Rather than viewing radicalization as mere disruption, this study demonstrates that it often represents a structured response to institutionalized violence embedded within state policies and economic frameworks (Galtung, 1969; Santos, 2002a,b).

Additionally, the eco-territorial turn proposed by Svampa (2019) emphasizes how struggles for land and natural resources are increasingly intertwined with demands for cultural recognition, political autonomy, and ecological sustainability. This framework is crucial for understanding how socio-environmental conflicts, such as the Razuhuillca strike, are embedded in broader genealogies of resistance against extractivist models imposed by the state.

The case of Huanta also invites reconsideration of the relationship between legality, legitimacy, and social mobilization. As Santos and Sena Martins (2020) argues, the coexistence of multiple normative systems within a single social space is not only a source of conflict but also a space for creativity and political agency. By asserting their right to self-determination and developing their own governance structures, Huanta’s communities engage in what Santos describes as the “sociology of absences”—making visible forms of knowledge and justice systematically ignored or delegitimized by official institutions.

Moreover, the findings challenge mainstream understandings of violence and radicalization in Latin America. While state narratives often frame radicalized protests as irrational or criminal, this study demonstrates that such actions are often deeply rooted in legitimate grievances and historical patterns of exclusion. As Escobar (2008) argues, recognizing alternative ontologies and epistemologies is essential to understanding the complexity of social mobilization in the region.

Ultimately, the case of Huanta illustrates that radicalization is not merely a destructive force but rather a creative strategy for asserting justice, dignity, and communal sovereignty. This dynamic, while evident in Huanta, is also reflected in broader struggles throughout Latin America, where grassroots actors continue to contest state power and reconfigure their relationships with territorial, political, and cultural authorities.

### Limits and internal contradictions of radicalization

While this study confirms that the radicalization of protest in Huanta is a legitimate and structured response to state exclusion, it also reveals internal tensions and contradictions that challenge its coherence and effectiveness. These limitations are essential to understand both the transformative potential and constraints of radicalization as a political strategy.

#### Human costs and ethical dilemmas

Radicalization has entailed significant human costs, particularly in the 2022 and 2024 protests, where several young participants lost their lives. Although community leaders often frame these deaths as sacrifices for a just cause, others express ethical concerns about the potential trauma and harm caused by persistent conflict. As one participant noted:

“We do not want our children to die for this struggle, but at the same time, we know that if we do not fight, everything is lost. It’s a constant dilemma.” (E10, Traditional community authority).

This testimony reveals not only the emotional toll of resistance but also the precarious conditions in which life itself becomes a field of dispute. As Mbembe (2003) argues, necropolitics refers to the exercise of power through the exposure of certain populations to death or abandonment. In the case of Huanta, the denial of access to justice, environmental protection, and basic guarantees reflects a form of governance that renders specific communities expendable. The dilemma captured in this quote—between defending life and risking it—epitomizes a necropolitical logic, in which communal survival is conditioned by systemic neglect and selective state absence.

This paradox illustrates the tension between the urgency of resistance and the ethical limits of mobilization. As Escobar (2008) argues, social movements must continually navigate the balance between militancy and preserving community well-being.

#### Fragmentation and strategic disagreements

The protests in Huanta reveal deep internal divisions, with differing views on whether confrontation or negotiation is the most effective strategy. While some actors advocate direct confrontation, others prefer dialogue, creating strategic disagreements that challenge unity. As one interviewee explained:

“It’s not that we all want to be there [in the protest], but sometimes it feels like an obligation. If you do not participate, you are seen as a traitor.” (E12, University student).

According to Habermas (1984), social movements’ legitimacy depends on their ability to foster dialogue and mutual understanding. When radicalization leads to coercion or exclusion of dissenting voices, it undermines the potential for deliberative and inclusive political processes.

#### Coercive cohesion and authoritarian tendencies

The emphasis on communal unity sometimes results in the suppression of dissent and the marginalization of alternative perspectives. While aiming to promote inclusion and justice, radicalization can inadvertently perpetuate exclusionary practices. This concern is evident in statements such as:

“If you do not march, if you do not speak out, they look at you like you are against them. It’s like you are no longer part of the community.” (E05, Territorial activist).

This testimony reveals an internal tension within resistance processes: the marginalization of those who propose alternative forms of participation. It exemplifies what Walsh (2007) describes as epistemic violence—the silencing or devaluation of non-dominant knowledge systems or expressions, even within community movements. In this case, dissenting views or non-confrontational strategies are delegitimized, reinforcing a hegemonic expectation of radicalism as the only valid mode of participation. As Rivera Cusicanqui (2010) argues, such internal exclusions mirror colonial hierarchies and risk reproducing the very patterns of domination that resistance movements seek to overcome.

This dynamic highlights the paradox of resistance: efforts to challenge oppressive structures can reproduce coercive practices within their own organizational frameworks. As Santos (2002a,b) notes, legal pluralism is not immune to internal contradictions and power imbalances, especially when unity becomes coercive rather than voluntary.

#### Exclusion of marginalized voices

The movement’s internal structures sometimes fail to adequately incorporate diverse perspectives, particularly those of women, youth, and elders advocating for non-confrontational approaches. This exclusion is not always intentional but often reflects broader patterns of social stratification and power dynamics within the community. As Rivera Cusicanqui (2010) argues, constructing alternative frameworks of governance requires actively challenging internal hierarchies and ensuring inclusivity.

#### Strategic inflexibility and risk of isolation

The adoption of confrontational tactics as the primary mode of resistance can also limit the movement’s ability to build alliances with external actors, such as NGOs, academic institutions, and international organizations. While aligning local struggles with transnational networks has strengthened the movement’s legitimacy, it has also created tensions over whether dialogue or confrontation is the appropriate path forward.

As Gudynas (2018) suggests, movements that become entrenched in specific strategies risk isolating themselves from broader political and institutional processes that could potentially advance their demands. This strategic inflexibility can undermine the adaptability and resilience of the movement.

## Conclusion

This study has examined the radicalization of protest in Huanta through three key dimensions: historical memory, state legitimacy crises, and socio-environmental conflicts in the Razuhuillca watershed. The findings confirm that mobilization in the region is not an isolated or circumstantial phenomenon but reflects a long-standing historical process of exclusion, structural violence, and struggles over territorial sovereignty.

The collective memory of past mobilizations plays a crucial role in legitimizing contemporary struggles. Protest repertoires are not static; they are reactivated and adapted to confront new challenges, reinforcing communal identities of resistance and validating direct action as a rational and structured response. This process of memory reactivation constitutes a form of resistance to symbolic violence, where communities reassert their historical dignity against state-imposed marginalization.

The burning of the Prosecutor’s Office in 2022 illustrates a profound legitimacy crisis, rooted in structural violence and the systematic denial of justice. The inability of the state to guarantee protection and address grievances has led communities to develop alternative systems of governance and popular justice, revealing a broader disconnection between institutional frameworks and the needs of marginalized populations.

The socio-environmental conflict in the Razuhuillca watershed further demonstrates how the defense of territory and natural resources becomes a fundamental axis of mobilization. Environmental struggles are not only material but also symbolic, encompassing broader demands for autonomy, cultural recognition, and the right to self-determination. The defense of water emerges as a response to necropolitical logics that expose certain populations to disposability and abandonment.

By integrating structural, symbolic, necropolitical, territorial, legal, and epistemic dimensions of violence, this study contributes to expanding new theoretical approaches to understanding how violence shapes grassroots mobilization and alternative governance in marginalized contexts. It offers a multidimensional framework that links historical grievances, contemporary socio-environmental struggles, and emerging forms of popular justice.

Overall, the radicalization of protest in Huanta emerges as a structured, coherent strategy rather than an isolated or irrational reaction. It is anchored in historical memory, fueled by cumulative experiences of exclusion and resistance, and strengthened by the defense of communal life against exclusionary and extractivist models.

Importantly, this study highlights that radicalization is not free from internal tensions. Strategic disagreements, ethical dilemmas, and risks of reproducing exclusionary practices within the movement itself reveal the complexity of collective resistance. Ensuring inclusivity, adaptability, and responsiveness to diverse perspectives will be essential for maintaining the legitimacy and sustainability of future mobilizations.

Future research should conduct comparative studies across different Andean and Latin American contexts to explore how radicalization processes evolve under varying political, economic, and cultural conditions. Examining the long-term impacts of protest criminalization and the development of new forms of communal organization will provide deeper insights into the dynamics of territorial resistance and political transformation.

## Data Availability

The raw data supporting the conclusions of this article will be made available by the authors, without undue reservation.
